# Pregnant and Nonpregnant Women in Cape Town, South Africa: Drug Use, Sexual Behavior, and the Need for Comprehensive Services

**DOI:** 10.1155/2011/353410

**Published:** 2011-04-06

**Authors:** Hendrée E. Jones, Felicia A. Browne, Bronwyn J. Myers, Tara Carney, Rachel Middlesteadt Ellerson, Tracy L. Kline, Winona Poulton, William A. Zule, Wendee M. Wechsberg

**Affiliations:** ^1^Substance Abuse Treatment Evaluations & Interventions Program, RTI International, 3040 Cornwallis Road, Research Triangle Park, NC 27709, USA; ^2^Alcohol and Drug Abuse Research Unit, Medical Research Council, P.O. Box 19070, Tygerberg, Cape Town 7505, South Africa

## Abstract

The multiple risks associated with methamphetamine use are of serious concern for women. These risks and consequences are magnified during pregnancy. This secondary analysis of a parent study compared 26 pregnant to 356 nonpregnant women in Cape Town, South Africa, on selected demographic, psychosocial, and HIV-risk domains to identify their treatment service needs. Proportionally, more pregnant than nonpregnant women are using methamphetamine, *P* = .01, although a very high rate of women used methamphetamine. Women reported similar monthly rates of sexual intercourse, but pregnant women were significantly less likely to report condom use, *P* < .0001, maintaining their risky behavior. Both groups reported elevated Center for Epidemiological Studies Depression Scale CES-D means, suggesting a need for depression treatment. Results demonstrate a pervasive need for women's comprehensive treatment, regardless of pregnancy status. Moreover, findings support the urgent need for women-focused and pregnancy-specific treatment services for methamphetamine use. Finally, a job-skills training/employment component focus is suggested.

## 1. Introduction

Cape Town, South Africa is experiencing a devastating level of methamphetamine use, with an estimated 7% of the adult population reporting the use of this drug [1, 2] (locally referred to as “tik”). While methamphetamine use is cause for concern in both sexes, South African social history and structure may provide a context that makes women, especially women of color who live in the township communities, Black (African and Xhosa-speaking) and Coloured (mixed racial ancestry and Afrikaans-speaking), vulnerable to intersecting risks [1–4]. If the woman remains untreated for her methamphetamine use, and then gets pregnant, the adverse consequences are likely to be exacerbated by continued drug use. 

Not only is dependence a danger for both pregnant and nonpregnant South African women who use methamphetamine, but use also increases risk of exposure to sexual risk behavior, sexual violence, and HIV, which co-occur with methamphetamine use [3–7]. 

Similar to the use of other substances, women typically begin using methamphetamine before they become pregnant. Once pregnant, they are often unable to stop using. In South Africa, women who live in poor communities do not usually seek antenatal care, are not very informed about drug treatment, are afraid of stigma from health care providers, and are especially vulnerable to drug-related sexual risk behaviors [7–9]. In addition to the maternal vulnerabilities associated with methamphetamine misuse, prenatal stimulant exposure has been associated with full-term birth but small gestational age [10], a risk factor for later developmental problems [11]. 

In Black and Coloured women in South Africa, the intersection of drug use, particularly methamphetamine use, HIV risk behaviors, and unplanned pregnancy must be addressed to improve the lives of these women and their children. Facing a methamphetamine epidemic, Cape Town is especially challenged with how to best reduce drug use in childbearing-age women who often do not enter formal drug treatment services. 

To develop effective women-specific drug treatment services designed to meet the particular needs of South African women, it is necessary to first examine the presenting issues that pregnant and nonpregnant drug users face. This secondary analysis of a parent study addresses this need by examining the baseline characteristics of pregnant and nonpregnant female drug users who signed informed consent to participate in an adapted evidence-based women-focused HIV behavioral intervention: the Western Cape Women's Health CoOp. Specifically, we compared pregnant and nonpregnant women on a priori selected baseline variables that were collected as a part of the main study. These baseline variables encompassed demographic, psychosocial, and HIV-risk domains to identify their shared and unique profiles of service needs.

## 2. Methods

### 2.1. Parent Study

The Western Cape Women's Health CoOp is an on-going community-based randomized control trial in Cape Town, South Africa that compares the effectiveness of a women-focused HIV behavioral intervention empowering women to reduce substance use, sexual risk, and victimization relative to two control conditions. The Western Cape Women's Health CoOp intervention was adapted from the original North Carolina Women's CoOp (an HIV intervention) that focused on empowering women to reduce their drug use and sexual risk behaviors [12]. The original Women's CoOp was culturally adapted for use among vulnerable sex-trading women in Pretoria, South Africa, to include a component on drug-related gender-based violence that focused on addressing sexual risk among vulnerable South African women [13]. Further changes in Cape Town for vulnerable women were piloted for group process [4].

### 2.2. Recruitment

Recruitment for study participation involved outreach that was conducted in targeted communities, namely, poor, mainly Black and Coloured communities surrounding the airport in Cape Town. A detailed sampling plan took into account the number of inhabitants of each community to help calculate the desired number of women to be recruited from that community. A targeted sampling plan was used to balance recruitment communities (i.e., outreach zones), and field staff worked in pairs to recruit study participants. Staff canvassed these streets and local hang-out spots frequented by women who use alcohol and other drugs and posted fliers and distributed leaflets to market the study. The field staff then approached and engaged women and verbally requested permission to administer a brief screening instrument to determine whether they were eligible to participate in the study. Recruitment began in September 2008 and ended in January 2011.

### 2.3. Participants

To be eligible, participants had to provide informed consent, be female and between the ages of 18 and 33, live in one of the target communities, report using at least two drugs once per week for the past 3 months, and report being sexually active with a male partner in the past month. At the time this smaller secondary study was conducted, a total of 382 participants were randomized into one of the study conditions, and their data are included in this analysis. Based on a pregnancy test administered at study entry, it was determined that 26 women were pregnant while 356 women were nonpregnant.

### 2.4. Measures

The Western Cape Women's Health CoOp Revised Risk Behavior Assessment (RRBA), adapted from the RRBA [14] was administered at study entry via computer-assisted participant interviews. The RRBA has 10 sections that contain questions about demographics and social characteristics, nutrition/health knowledge, alcohol use, drug use, drug injecting, sexual behavior, power and empowerment, conflict and victimization (stigma and vulnerability), obstetrical/physical/mental health, and HIV. The 20-item Center for Epidemiological Studies Depression Scale (CES-D) was used to determine the depressive symptom intensity experienced by these women [15, 16]. The CES-D also has been validated in Cape Town, South African women. Scores range from 0 to 60. A score of 16 in the United States and in South Africa indicates high risk for clinical depression [16, 17]. Pregnancy and self-reported drug use were confirmed with urine testing.

### 2.5. Statistical Analysis

Welch's *t* test assuming unequal population variances and Satterthwaite's approximation for degrees of freedom was employed to analyze the continuous outcome measures. Because the sample sizes in the cells was disproportionate, and sometimes small for the pregnant women, the cross-tabulation tables were therefore sparse, so exact test statistics were used to conduct the goodness-of-fit tests for the categorical outcomes.

## 3. Results

### 3.1. Participant Characteristics

The total sample (Table 1) was predominantly Coloured (63.6%), in their early 20s, single, and having completed, on average, less than the 12 years required for graduation from high school. While few of the women were employed (9.7%), more than three-quarters indicated that they have skills for employment. They had, on average, two children living with them. More than 1/3 had spent time in prison. Finally, the elevated mean score on the CES-D would strongly suggest that a substantial percentage of these women were currently experiencing significant depressive symptoms. 

#### 3.1.1. Alcohol and Drug Use

Table 1 shows that past-month drug use differed by pregnancy status only for methamphetamine (“tik”) use, with a larger proportion of pregnant than nonpregnant women reporting methamphetamine use (92.3% versus 66.9%; *P* = .01). Use of alcohol (91.7%) and marijuana, or “dagga,” (73.6%) was reported by an overwhelming majority of both groups. In contrast, both groups reported infrequent use of most other drugs, including heroin and cocaine. Almost two-thirds of the sample indicated they believed they had a drug problem; only a small percentage of the sample reported they had an alcohol problem. These statements appear equally true for both the pregnant as well as the nonpregnant sample.

#### 3.1.2. Sexual Behavior

Almost all participants had a main sex partner (Table 1).  Table 2 reveals that more than half the sample had used drugs or alcohol before or during sex, and that more than half had traded sex for money, and more than one-third had traded sex for drugs in the past 30 days. The mean number of sex partners in the past 30 days was quite low (1.29 (SD = 2.0)), although more than two-thirds indicated they had engaged in sex with a casual partner. While over 90% of both groups reported having sex with their main partner in the past month, pregnant women reported fewer times of condom use in the past month than did nonpregnant women (.26 versus 2.04, *P* < .0001). 

In the pregnant sample, only 34.6% of women received prenatal care, although 73.1% sought such care (Table 1). However, it should be noted that a self-report of receipt of prenatal care could include as little as setting up a delivery date. More than 50% of the women indicated that they had reduced or stopped their alcohol, dagga, and methamphetamine use; however, clinically concerning percentages of women were still using these substances during pregnancy (Table 1). This statement is particularly true for methamphetamine, for which more than 30% of the pregnant sample reported that their use of methamphetamine was the same frequency or more frequent than before they became pregnant.

#### 3.1.3. Needs Assessment

Table 3 indicates that the most requested services included services in the areas of employment (86.7%), financial assistance (82.5%), and housing (68.3%); medical (53.4%), transportation (48.4%), alcohol/drug services (47.6%), school (45.8%), and HIV/STD testing (42.9%) all hover around 50%. Approximately one-quarter of the sample expressed a need for sexual abuse and/or legal assistance services. Mental health assistance was least requested (19.1%). Finally, it should be noted that a greater proportion of pregnant than nonpregnant women wanted educational assistance (65.4% versus 44.4%; *P* = .04).

## 4. Discussion

The study has several major findings. First, there is the concerning rate of methamphetamine use among pregnant women compared with nonpregnant women. The fact that over 90% of the pregnant women reported recently using methamphetamine is consistent with another recent study in Cape Town, which showed that among a group of pregnant women who smoked tobacco, up to 78% reported methamphetamine use [18]. These results are also consistent with other reports of high rates of methamphetamine use observed in Cape Town among the general population, its women, and its out-of-school young women between 13 to 20 years old [4, 8]. Taken together, the past and current data highlight the urgent need to develop and implement effective women-focused interventions to reduce this epidemic. The high rate of methamphetamine use among pregnant women may also be a reflection of the relationship between methamphetamine use and sexual risk behavior, which can result in pregnancy. On a positive note, the majority of the pregnant women made attempts to reduce their drug use after learning that they were pregnant, which is an encouraging sign. However, more than 30% of the pregnant sample either used at the same level or increased use of methamphetamine. Given the potentially adverse impact of stimulants on fetal brain development, particularly in the context of multiple environmental risk factors, there is a clear need for prevention initiatives in the Western Cape of South Africa, similar in scope and focus to efforts for informing the public about fetal alcohol spectrum disorders. 

The second major finding of this secondary analysis is the pervasive need for comprehensive treatment for pregnant Black and Coloured women in Cape Town, South Africa. Although these women have myriad needs, the pregnant women in this sample necessarily required medical and obstetrical care. The literature from South Africa suggests that over the 16 years since National Health System has provided free antenatal and intrapartial care to all uninsured women, many women have not been accessing or have been underutilizing care before delivery [19]. The reasons underlying this underutilization are complex and include structural barriers (e.g., inconvenient hours of clinic operation, inability to take time off from work, too long a wait to be seen, lack of child care, and/or transportation); relationship issues (e.g., unstable relationship with the father of the baby); psychological factors (e.g., unwanted pregnancy, pregnancy denial); lack of education as to the need for prenatal health care; negative interactions with the medical professionals [20–24]. The current findings, in context with the prior literature, suggest that women want medical care as well as drug and alcohol treatment, yet interventions must be implemented where they can be reached and at multiple levels to concurrently address the structural barriers to care, improve the actual care these women receive, and reduce the perceived and actual stigma and discrimination the women feel and encounter.

The third finding of note is that the pregnant women had a lower rate of condom use than did the nonpregnant women. This result suggests that a significant proportion of methamphetamine-using Black and Coloured women consider condoms to serve the primary role of birth control rather than of disease prevention. If this conclusion is supported by future research, community outreach efforts need to be made to educate this high-risk population regarding the crucial role that condoms can play in HIV/sexually transmitted infection prevention. 

Finally, it is notable that these women made a clear distinction between mental health services and alcohol/drug services. Fewer than 1 in 5 women expressed a need for mental health services, despite the CES-D mean score being almost twice that of the clinical cutoff used previously in low-income US and South African women. Although increased depressive symptoms are associated with being of color, lower educational attainment, and low income; having substandard living conditions; living in stressful neighborhoods; possibly, lacking support of a partner [17], this finding underscores the need for intervention to ameliorate the symptoms of depression while concurrently treating drug addiction.

In contrast to the low self-reported need for mental health services, almost 1 in 2 women expressed a need for alcohol/drug services. Moreover, the most pressing needs expressed were for economic support, with more than three-quarters of the women wanting employment and financial aid; housing only slightly trailed the former two needs. These conclusions appear to be equally valid for the pregnant women as for the nonpregnant women.

## 5. Study Limitations

As with all studies, the present study has its limitations. First, it involved a preliminary and secondary analysis to the aims from ongoing larger Western Cape Women's Health CoOp project, which has a focus on HIV prevention that is different from the present project. Thus, the inclusion and exclusion criteria of the parent project may have adversely impacted the ability to recruit a representative sample of Black and Coloured South African women. Second, the extent to which these results generalize to the larger population of Black and Coloured South African women is unknown. Third, because the primary focus of the parent project was not pregnancy and substance use, the number of respondents who were pregnant was relatively low in comparison to the size of the nonpregnant sample. Fourth, the RRBA was focused on collecting a wide variety of information, not all of which was maximally relevant to pregnant women. Despite the limitations, the findings provide considerable initial information on the similarities and unique issues for pregnant and nonpregnant Black and Coloured South African women.

## 6. Conclusions

Study findings strongly support two conclusions. First, the widespread use of methamphetamine in pregnant Black and Coloured South African women indicate an urgent need for the development and implementation of comprehensive treatment programs to address methamphetamine use (as well as other co-occurring substance use) in these women. Second, findings suggest that both common and unique issues between pregnant and nonpregnant women must be addressed when developing and adapting comprehensive treatments for substance-using Black and Coloured South African women of childbearing age. Notably, the dismal circumstances that impact the health and well-being of many of these women (and their children) are unlikely to change until they are provided with women-centered medical and obstetrical care and drug treatment. Moreover, programs should include employment or job-skills training so that these women can break the cycle of trading sex for money and drugs.

## Figures and Tables

**Table 1:** Demographic and Background Characteristics of Pregnant and Nonpregnant Women (*N* = 382).

Measure	Total sample (*N* = 382)	Pregnant women (*n* = 26)	Nonpregnant women (*n* = 356)	Test statistic (*t*(*df*) or *χ* ^2^(*df*))	*P*
	Mean (SD) or *n* (%) or *n*/*N* (%)				

*Demographics and Social characteristics*					

Age	23.1 (4.2)	22.7 (3.6)	24.3 (4.3)	*t*(30) = −2.22	.03
Race					
Black	139 (36.4%)	5 (19.2%)	134 (37.6%)	*χ* ^2^(1) = 3.55	.09
Coloured	243 (63.6%)	21 (80.8%)	222 (62.4%)		
Mean years of education completed	9.3 (1.9)	9.0 (2.2)	9.3 (1.9)	*t*(28) = −0.69	.49
Marital status					
Single	353 (92.4%)	24 (92.3%)	329 (92.4%)	*χ* ^2^(1) = 0.00	1.0
Married	29 (7.6%)	2 (7.7%)	27 (7.6%)		
Have a main sexual partner	359 (94.0%)	24 (92.3%)	335 (94.1%)	*χ* ^2^(1) = 0.14	.66

*Economic status*					
Employed	37 (9.7%)	0 (0.0%)	37 (10.4%)	*χ* ^2^(1) = 3.00	.16
Have skills for employment	290 (75.9%)	20 (76.9%)	270 (75.8%)	*χ* ^2^(1) = 0.02	1.0
Mean number of children living with participant	2.0 (1.7) (*n* = 351)	2.1 (1.1) (*n* = 17)	2.0 (1.7) (*n* = 325)	*t*(20) = 0.05	.96

*Legal history*					
Ever spent time in prison	142 (37.2%)	9 (34.6%)	133 (37.4%)	*χ* ^2^(1) = 0.08	.84

*Violence exposure *					
Ever been physically hurt	171 (44.8%)	8 (30.8%)	163 (45.8%)	*χ* ^2^(1) = 2.21	.16
Ever forced to perform sexual acts	89 (23.3%)	6 (23.1%)	83 (23.3%)	*χ* ^2^(1) = 0.001	1.0

*Risk factors for substance abuse*					
Depressive symptom severity: mean CES-D score in past week	28.4 (12.2)	29.7 (9.9)	29.4 (12.2)	*t*(31) = 0.16	.87
Family members use drugs and/or alcohol too much	289 (75.7%)	22 (84.6%)	267 (75.0%)	*χ* ^2^(1) = 1.22	.35
Maternal history of alcohol use	183 (47.9%)	12 (46.2%)	171 (48.0%)	*χ* ^2^(1) = 0.03	1.0
During the past 30 days, did your main partner get drunk	211/359 (58.8%)	13/24 (54.2%)	198/335 (59.1%)	*χ* ^2^(1) = 0.23	.67
During the past 30 days, did your main partner use drugs	232/359 (64.6%)	13/24 (54.2%)	219/335 (65.4%)	*χ* ^2^(1) = 1.23	.28

*Alcohol and drug use and drug injecting*					
Methamphetamine (tik) use	262 (68.6%)	24 (92.3%)	238 (66.9%)	*χ* ^2^(1) = 7.29	.01
Drink alcohol	341/372 (91.7%)	23/26 (88.5%)	318/346 (91.9%)	*χ* ^2^(1) = 0.38	.47
How often do you drink alcohol	(*n* = 372)	(*n* = 26)	(*n* = 346)		
Never	31 (8.3%)	3 (11.5%)	28 (8.1%)		
Monthly or less	90 (24.2%)	4 (15.4%)	86 (24.9%)		
2–4 times per month	101 (27.2%)	12 (46.2%)	89 (25.7%)	*χ* ^2^(4) = 6.26	.14
2–3 times per week	114 (30.7%)	5 (19.2%)	109 (31.5%)		
4 or more times per week	36 (9.7%)	2 (7.7%)	34 (9.8%)		
Marijuana (dagga) use	281 (73.6)	17 (65.4%)	264 (74.2%)	*χ* ^2^(1) = 0.96	.36
Rock use	12 (3.1%)	1 (3.8%)	11 (3.1%)	*χ* ^2^(1) = 0.05	.58
Cocaine use	2 (0.5%)	0 (0%)	2 (0.6%)	*χ* ^2^(1) = 0.15	1.0
Heroin use	13 (3.4%)	0 (0%)	13 (3.7%)	*χ* ^2^(1) = 0.98	1.0
Mandrax (white pipe) use	98 (25.7%)	8 (30.8%)	90 (25.3%)	*χ* ^2^(1) = 0.38	.50
Ever injected	3 (0.8%)	0 (0%)	3 (0.84%)	*χ* ^2^(1) = 0.22	1.0
Participant thinks she has an alcohol problem	52 (13.6%)	2 (7.7%)	50 (14.0%)	*χ* ^2^(1) = 0.83	.55
Participant thinks she has a drug problem	244 (63.9%)	16 (61.5%)	228 (64.0%)	*χ* ^2^(1) = 0.07	.83

*Drug treatment history *					
Ever been to drug treatment	34 (8.9%)	3 (11.5%)	31 (8.7%)	*χ* ^2^(1) = 0.24	.72

*Reasons for not entering drug Treatment*					
Drug treatment does not work	100/348 (28.7%)	7/23 (30.4%)	93/325 (28.6%)	*χ* ^2^(1) = 0.03	.82
Participant does not know where to go for treatment	95/349 (27.2%)	2/23 (8.7%)	93/326 (28.5%)	*χ* ^2^(1) = 4.27	.05
Lack the money to pay for treatment	69/349 (19.8%)	2/23 (8.7%)	67/326 (20.6%)	*χ* ^2^(1) = 1.9	.28

*Nutrition*					
Frequency of going without food					
Never	127 (33.3%)	6 (23.1%)	121 (34.0%)	*χ* ^2^(3) = 1.36	.64
Less than once a month	87 (22.8%)	7 (26.9%)	80 (22.5%)		
Less than once a week	73 (19.1%)	6 (23.1%)	67 (18.8%)		
Every week	95 (24.9%)	7 (26.9%)	88 (24.7%)		

*Obstetrical status*					
Now pregnant	26 (6.8%)				
Seek prenatal care		19 (73.1%)			
Received prenatal care		9 (34.6%)			
Use of methamphetamine (tik) while pregnant					
Never used		2 (7.7%)			
Stopped		0 (0%)			
Reduced		16 (61.5%)			
Same		7 (26.9%)			
Increased		1 (3.9%)			
Use of alcohol while pregnant					
Never used		2 (7.7%)			
Stopped		9 (34.6%)			
Reduced		10 (38.5%)			
Same		5 (19.2%)			
Use of marijuana (dagga) while pregnant					
Never used		5 (19.2%)			
Stopped		3 (11.5%)			
Reduced		13 (50.0%)			
Same		4 (15.4%)			
Increased		1 (3.9%)			

*Notes.* Probability values for *χ* ^2^ tests of significance are based on exact methods. Percentages are within their respective sample.

**Table 2:** Sexual behavior of pregnant and nonpregnant women (*N* = 382).

Measure	Total sample (*N* = 382)	Pregnant women (*n* = 26)	Nonpregnant women (*n* = 356)	Test statistic (*t*(*df*) or *χ* ^2^(*df*))	*P*
Last sex was with a man	374 (97.9%)	26 (100%)	348 (97.8%)	*χ* ^2^(1) = 0.60	1.0
At last sex act, participant used drugs or alcohol use before or during sex	202 (52.9%)	11 (42.3%)	191 (53.7%)	*χ* ^2^(1) = 1.25	.31
Mean age at first vaginal sex	16.2 (2.73)	16.2 (1.63)	16.3 (2.95)	*t*(38) = −0.19	.85
First vaginal sex was willing	307 (80.4%)	22 (84.6%)	285 (80.1%)	*χ* ^2^(1) = 0.32	.80
Condom used first time	117 (30.6%)	12 (46.2%)	105 (29.5%)	*χ* ^2^(1) = 3.17	.08
Past 30 day sex with main partner	349/359 (97.2%)	24/24 (100%)	325/335 (97.0%)	*χ* ^2^(1) = 0.74	1.0
Mean of sex partners in past 30 days	1.29 (2.0)	1.11 (0.4)	1.36 (2.4)	*t*(199) = −1.57	.12
Mean times of sex with main partner in past 30 days	8.09 (6.90)	6.04 (7.96)	8.24 (6.81)	*t*(25) = −1.29	.25
Mean times of condom use with sex with main partner in past 30 days	1.93 (4.29)	0.26 (0.92)	2.04 (4.40)	*t*(129) = −5.75	<.0001
Drugs and alcohol leads to sex risk	84 (22.0%)	7 (26.9%)	77 (21.6%)	*χ* ^2^(1) = 0.40	.62
Trading sex for money in past 6 months	31/58 (53.5%)	3/5 (60%)	28/53 (52.8%)	*χ* ^2^(1) = 0.09	1.0
Trading sex for drugs in past 6 months	23/58 (39.7%)	1/5 (20.0%)	22/53 (41.5%)	*χ* ^2^(1) = 0.88	.64
Last time had sex:					
48 hrs	123 (32.2%)	10 (38.5%)	113 (31.7%)		
3–7 days	157 (41.1%)	11 (42.3%)	146 (41.0%)	*χ* ^2^(2) = 0.93	.89
In last 8–30 days	102 (26.7%)	5 (19.2%)	97 (27.3%)		
At last sexual encounter the woman participant was willing	368 (96.3%)	26 (100%)	342 (96.0%)	*χ* ^2^(1) = 1.06	.61

*Note.* Probability values for *χ* ^2^ tests of significance are based on exact methods.

**Table 3:** Areas in which participants expressed a need for a social service (*N* = 382).

Measure	Total sample (*N* = 382)	Pregnant women (*n* = 26)	Nonpregnant women (*n* = 356)	*χ* ^2^	*P*

*Service area*	*n* (%)				
Employment	331 (86.7%)	21 (80.8%)	310 (87.1%)	0.83	.37
Financial assistance	315 (82.5%)	19 (73.1%)	296 (83.2%)	1.70	.19
Housing	261 (68.3%)	17 (65.4%)	244 (68.5%)	0.11	.83
Medical	204 (53.4%)	15 (57.7%)	189 (53.1%)	0.21	.69
Transportation	185 (48.4%)	12 (46.2%)	173 (48.6%)	0.06	.84
Alcohol/drug services	182 (47.6%)	10 (38.5%)	172 (48.3%)	0.94	.42
School	175 (45.8%)	17 (65.4%)	158 (44.4%)	4.31	.04
HIV/STD testing	164 (42.9%)	8 (30.8%)	156 (43.8%)	1.68	.22
Child care	113 (29.6%)	7 (26.9%)	106 (29.8%)	0.09	.83
Sexual abuse	99 (25.9%)	4 (15.4%)	95 (26.7%)	1.61	.25
Legal assistance	93 (24.4%)	5 (19.2%)	88 (24.7%)	0.40	.64
Mental health	73 (19.1%)	2 (7.7%)	71 (19.9%)	2.35	.19

*Notes. d* *f* = 1 for all *χ* ^2^ tests of significance. Probability values are based on exact methods.
